# Correction: The unmet need for COVID-19 treatment in immunocompromised patients

**DOI:** 10.1186/s12879-023-08034-0

**Published:** 2023-01-31

**Authors:** Alessandra D’Abramo, Serena Vita, Emanuele Nicastri

**Affiliations:** National Institute for Infectious Diseases “Lazzaro Spallanzani” IRCCS, Via Portuense 292, 00149 Rome, Italy

**Correction: BMC Infectious Diseases (2022) 22:930** 10.1186/s12879-022-07918-x

Following publication of the original article [1], the authors identified an error in the author names of Alessandra D’Abramo, Serena Vita, Emanuele Nicastri. The given name and family name were erroneously transposed. This extended to the Author contributions section.

- **The incorrect author names are**: D’Abramo Alessandra, Vita Serena, Nicastri Emanuele.
- **The incorrect Author contributions:** DA, VS and NE performed literature search, drafted and completed the manuscript. All authors read and approved the final manuscript.
- **The correct author names are:** Alessandra D’Abramo, Serena Vita, Emanuele Nicastri.
- **The correct Author contributions:** AD, SV and EN performed literature search, drafted and completed the manuscript. All authors read and approved the final manuscript.

The author group and Author contributions have been updated above and the original article [1] has been corrected.
